# Using UPLC-QTOF-MS Method to Analyse the Spot Constituents of the Thin Layer Chromatograms for Chuzhou* Stemona sessilifolia* (Miq.) Miq.

**DOI:** 10.1155/2019/3645793

**Published:** 2019-04-01

**Authors:** Ya-Zhong Zhang, Ye Tao, Ai-Zong Shen

**Affiliations:** ^1^Anhui Institute of Food and Drug Control, Hefei 230051, China; ^2^Anhui Provincial Hospital of the First Affiliated Hospital of University of Science and Technology of China, Hefei 230001, China

## Abstract

A simple, rapid, and highly sensitive analytical method was established for identification of constituents in the spot of the thin layer chromatogram of Chuzhou* Stemona sessilifolia* (Miq.) Miq. (Chuzhou* S. sessilifolia*) with ultra performance liquid chromatography-quadrupole time-of-flight mass spectrometry (UPLC-QTOF-MS). The technology was applied to systematically analyze and detect the targeted spots. Compared with the fragmentation behaviors of more than thirty reference constituents, the possibly existing compounds of the target spots were identified or tentatively identified by their exact masses and diagnostic fragment ions. Finally, the four clear spots of the thin layer chromatograms of Chuzhou* S. sessilifolia* were screened and identified of possible molecular formula and structures.

## 1. Introduction

Stemona Root is the dried root tuber of* Stemona sessilifolia* (Miq.) Miq. (*S. sessilifolia*),* Stemona japonica* (Bl.) Miq., or* Stemona tuberose* Lour. (Fam. Stemonaceae). The herbal drug is collected in summer or autumn and then removed of rootlet, washed clean, treated with boiling water for a moment or steamed until the center of the cut section surface is devoid of a white core, and finally dried in the sun [1].

*S. sessilifolia* is one of the three largest sources of Stemona Root. Its flavor is mild warm, sweet, and bitter. It belongs to lung meridian. The therapeutic action of* S. sessilifolia* includes moistening the lung, direct qi downward to suppress cough, and killing worms and lice [2–8].

*S. sessilifolia* is planted in several provinces such as Anhui, Henan, and Jiangsu in China [9–11]. However, the genuine production district of* S. sessilifolia* is regarded to be Chuzhou county of Anhui Province [12–14] and is thus known as Chuzhou* S. sessilifolia*[7, 15].

We collected 10 batches of* S. sessilifolia* samples from districts all over the country of China, with seven batches from Chuzhou county.

On the basis of a large number of references, we have carried out a lot of preliminary research work on* S. sessilifolia* [16–18] and established the thin layer chromatography method used to confirm that protostemonine is just the index chemical composition.

From the thin layer chromatogram, we found the identical spots which exist in the products from major producing areas of Chuzhou county and is, called by us, the geographical indication spot. Herein, we used UPLC-QTOF-MS method to identify the constituents of the geographical indication spot and other three clear spots of Chuzhou* S. sessilifolia*. Moreover, we inferred the possible molecular formula and structures of the constituents of the four spots.

## 2. Materials and Methods

### 2.1. Instruments

The instruments used for this study include Automatic TLC Sampler 4 (Switzerland CAMAG company), Camag TLC Visualizer (Switzerland CAMAG company), Waters Acquity UPLC™, Xevo G2 QTOF mass spectrometer, Waters Marker Lynx 4.1 chromatographic workstation, Electronic analytical balance (BS210S, Sartorius), Numerical control ultrasonic cleaner (Kunshan Ultrasonic Instrument Co., Ltd.), Merck SG60 F254 prefabricated slab, and twin trough glass chamber.

### 2.2. Chemicals and Reagents

Methanol and acetonitrile of HPLC-grade were purchased from Fisher Chemical. Ether, ethanol, cyclohexane, ethyl acetate, acetone, and ammonium hydroxide solution (25%, v/v) were of analytical purity and purchased from Sinopharm Chemical Reagent Co., Ltd.

Ammonium carbonate, guaranteed reagent, was purchased from Sinopharm Chemical Reagent Co., Ltd. Ultrapure water was self-made with the Milli-Q water purification system (Millipore, Milford, MA).

And 5% (w/v) sodium nitrite in 70% (v/v) ethanol solution, the dilute bismuth potassium iodide solution, and the reference substance of protostemonine (purity >98%) were prepared in our lab.

A total of 10 batches of* S. sessilifolia* samples were collected from Anhui and Henan provinces and authenticated by Prof. Zhang Yazhong (Department of Chinese Medicine Tests, Anhui Institute for Food and Drug Control, China).

### 2.3. UPLC-QTOF-MS Conditions

Chromatographic analyses were developed on Waters Acquity UPLC™.

The chromatographic separation was achieved on Acquity UPLC BEH Shield RP C18 column (2.1 × 150 mm, 1.7*μ*m) at 30°C with a flow rate of 0.4 ml/min. Mobile phases of A and B were acetonitrile and 0.25 mM ammonium carbonate solution. The gradient elution programs were as follows: 0 -10 min, 85-20% B; 10-10.50 min, 20-85% B; and 10.5-12.00 min, 85% B. The detector was PDA, and injection volume was 5 *μ*l.

The mass spectrometer analysis was performed on Water Xevo G2 QTOF/MS (Waters), operating in the positive electrospray ionization mode, and collecting data in the full-scan mode in the mass range m/z 80-1000 with a 0.2 s scan time.

The other optimal parameters employed were as follows: the capillary voltage was 3.0 kV; the cone voltage was 20-60 V; the source temperature was 150°C; the desolvation temperature was 450°C; the desolvation gas flow was 600 L/h. All data were recorded and processed by Waters Marker Lynx 4.1 Chromatographic workstation.

### 2.4. Sample Preparation

Pretreatment for the powdered sample with reagent and preparation of sample solution was carried out according to a previous report [19].

### 2.5. Reference Standard Preparation

Protostemonine was dissolved with ethanol [19].

### 2.6. Thin Layer Chromatography

According to the preliminary exploration of the developing solvent system, the TLC plate, the mobile phase, sample volume, spraying reagent, and observation condition were established [19].

### 2.7. Pretreatment of the Thin Layer Plate Spot

The corresponding position of the four spots was in Figure 2. The four corresponding spots were scraped out with a knife and dissolved in 5 ml of methanol, followed by sonication (360 W) for 10 min and then filtration to obtain the sample solutions. For control, the blank of silica gel G plate was also scraped out and treated in the same way as the sample solutions.

## 3. Results and Discussion

### 3.1. Thin Layer Chromatogram of Samples

The mobile phase was acetate-acetone-ammonium hydroxide solution (the upper layer) (6:4:4:1) and the spotting was carried out with 5 *μ*l sample volume.

**Figure 1:**
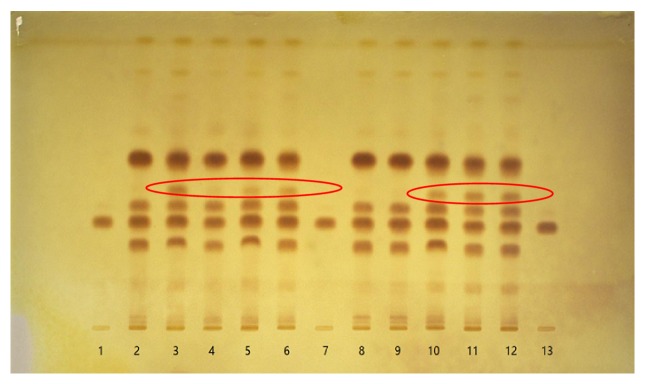
TLC diagram of roots of S*temona sessilifolia* (Miq.) Miq. 1,7,13: protostemonine 2-6,8-12: BB-01-001-BB-01-010.

Under the above method, 10 batches of samples were separated without any tailing and diffuseness (Figure 1).

The thin layer chromatographic behaviors of the 10 batches of samples were similar. It indicated that the species of alkaloids of samples from different producing areas were consistent. Samples numbered 3,4,5,6,10,11,12 showed one more spot than those numbered 2,8,9, as marked in red circles. Since samples numbered 3,4,5,6,10,11,12 were from Chuzhou county, we inferred that the extra spot was the geographical indication spot of Chuzhou* S. sessilifolia*.

### 3.2. Selection of Thin Layer Chromatography Spots

**Figure 2:**
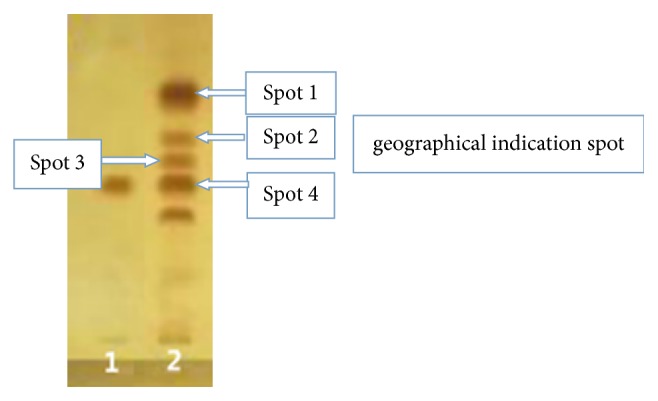
Spot location.

We selected four spots from the sample of Chuzhou* S. sessilifolia*, including the geographical indication spot corresponding to protostemonine (Figure 2).

### 3.3. Composition of Spots Analysis with UPLC-QTOF-MS Method

**Figure 3:**
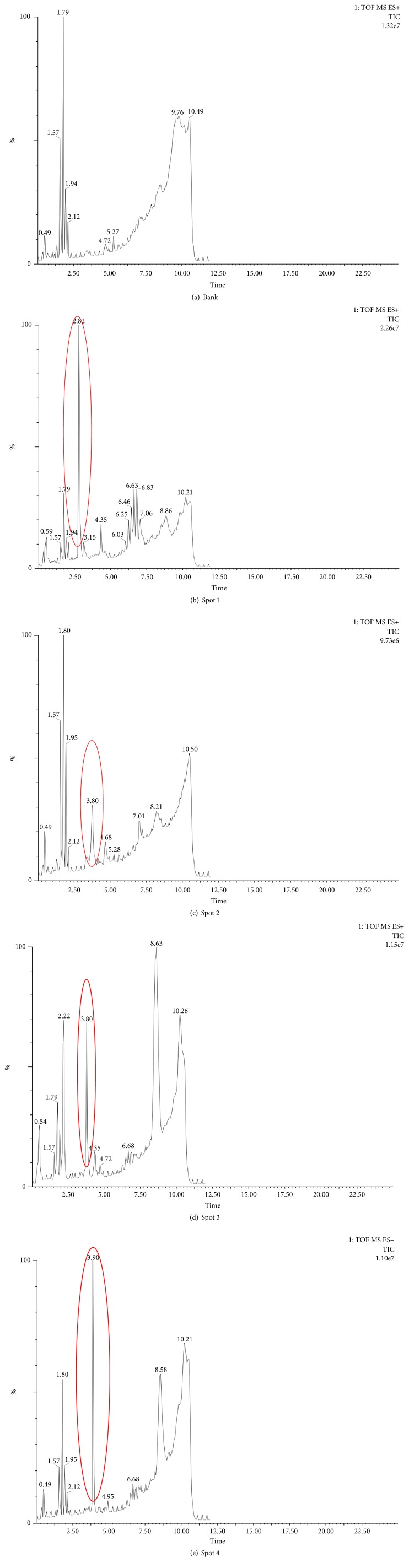
Total ion chromatograms of blank, spot 1, spot 2, spot 3, and spot 4 by UPLC-QTOF-MS in positive mode.

Injection of 5 ul of the blank or sample solution into the UPLC- QTOF-MS system resulted in total ion chromatograms of four spots by UPLC-QTOF-MS, as shown in Figure 3. Comparing the retention time, one characteristic chromatographic peak was detected in the total ion chromatogram of each thin layer chromatography spot, as marked by the red circle.

Then we analyzed the fragment ion spectrogram of the four spots.

**Figure 4:**
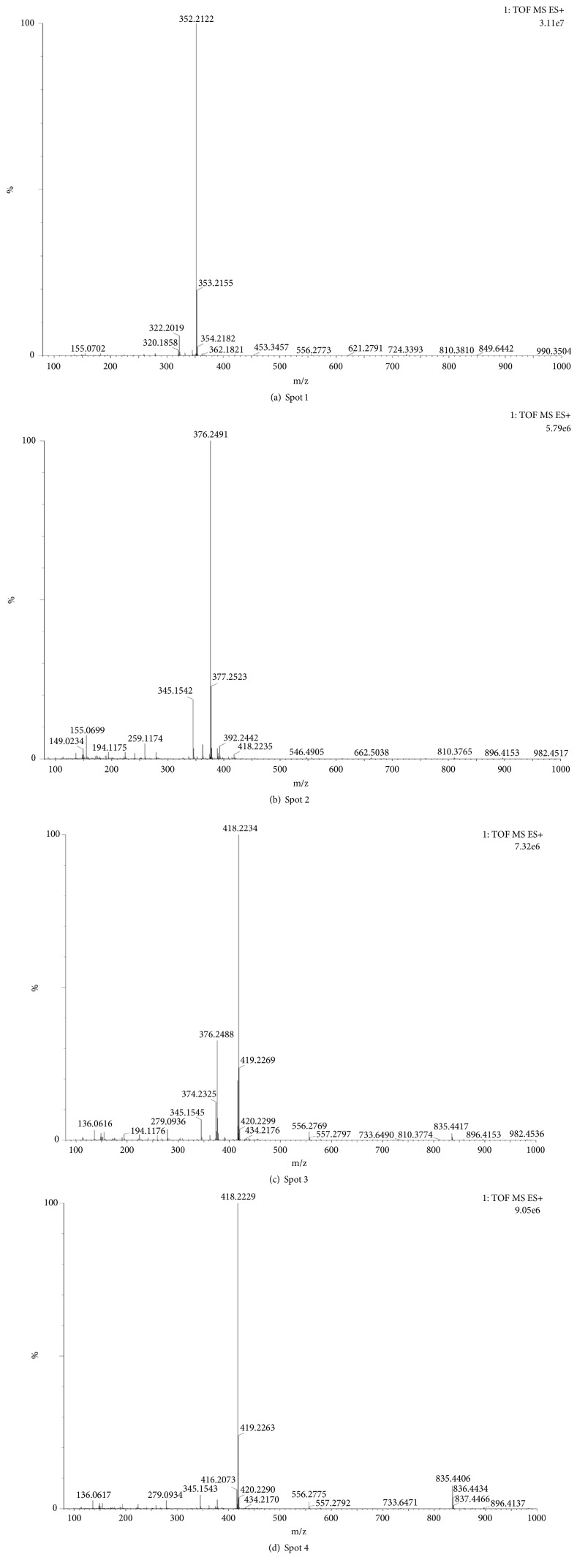
MS spectra of four spots.

The detailed mass spectrograms were listed in Figure 4.

The main constituents of four spots were tentatively confirmed in comparison to the known available reference standards with the mass spectra. The detailed structural information of four spots was listed in Table 1.

## 4. Conclusion

In conclusion, we identified the geographical indication spot of Chuzhou* S. sessilifolia* by establishing the thin layer chromatogram of* S. sessilifolia*. In addition, a reliable and sensitive well-developed method based on UPLC-QTOF-MS in a positive ion mode was used for analyzing and inferring compounds of the geographical indication spot and three other clear spots of Chuzhou* S. sessilifolia*. It may provide a certain basis for demonstrating the particular geoherbalism of Chuzhou* S. sessilifolia*.

## Figures and Tables

**Table 1:** Identification of compounds in four spots.

NO	t_R_	Production	m/z	Formula	Compound
1	2.848	[M + H]^+^	352.2122	C_19_H_29_NO_5_	stemonidine
2	3.906	[M + H]^+^	376.2491	C_22_H_33_NO_4_	sessilistemonamine D
3	3.774	[M + H]^+^	418.2234	C_23_H_31_NO_6_	isoprotostemonine
4	3.929	[M + H]^+^	418.2229	C_23_H_31_NO_6_	protostemonine

## Data Availability

Previously reported data were used to support this study and are available at [DOI: 10.1556/1006.2015.28.6.5]. These prior studies (and datasets) are cited at relevant places within the text as reference [19].
